# Brain Signatures of Embodied Semantics and Language: A Consensus Paper

**DOI:** 10.5334/joc.237

**Published:** 2023-10-10

**Authors:** Laura Bechtold, Samuel H. Cosper, Anastasia Malyshevskaya, Maria Montefinese, Piermatteo Morucci, Valentina Niccolai, Claudia Repetto, Ana Zappa, Yury Shtyrov

**Affiliations:** 1 Institute for Experimental Psychology, Department for Biological Psychology, Heinrich-Heine University Düsseldorf, Germany; 2 Institute of Cognitive Science, University of Osnabrück, Germany; 3 Centre for Cognition and Decision making, Institute for Cognitive Neuroscience, HSE University, Russian Federation; 4 Potsdam Embodied Cognition Group, Cognitive Sciences, University of Potsdam, Germany; 5 IRCCS San Camillo Hospital, Venice, Italy; 6 Basque Center on Cognition Brain and Language, Donosti, Spain; 7 Institute of Clinical Neuroscience and Medical Psychology, Medical Faculty, Heinrich-Heine University Düsseldorf, Germany; 8 Department of Psychology, Università Cattolica del Sacro Cuore, Milan, Italy; 9 Laboratoire parole et langage, Aix-Marseille Université, Aix-en-Provence, France; 10 Center of Functionally Integrative Neuroscience, Department of Clinical Medicine, Aarhus University, Denmark

**Keywords:** embodied cognition, EEG, priming, language learning, semantic processing, virtual reality

## Abstract

According to embodied theories (including embodied, embedded, extended, enacted, situated, and grounded approaches to cognition), language representation is intrinsically linked to our interactions with the world around us, which is reflected in specific brain signatures during language processing and learning. Moving on from the original rivalry of embodied vs. amodal theories, this consensus paper addresses a series of carefully selected questions that aim at determining *when* and *how* rather than *whether* motor and perceptual processes are involved in language processes. We cover a wide range of research areas, from the neurophysiological signatures of embodied semantics, e.g., event-related potentials and fields as well as neural oscillations, to semantic processing and semantic priming effects on concrete and abstract words, to first and second language learning and, finally, the use of virtual reality for examining embodied semantics. Our common aim is to better understand the role of motor and perceptual processes in language representation as indexed by language comprehension and learning. We come to the consensus that, based on seminal research conducted in the field, future directions now call for enhancing the external validity of findings by acknowledging the multimodality, multidimensionality, flexibility and idiosyncrasy of embodied and situated language and semantic processes.

The embodiment and grounded frameworks (Barsalou, 2008) propose an alternative view of language processing to the earlier amodal approach (Fodor, 1985). Amodal approaches assume word meanings are symbolic and thereby separated from sensorimotor substrates and individual experience. For this consensus paper, we grouped the so-called *4Es* (that is, *embodied, embedded, extended, enacted cognition*) as well as *grounded cognition* under the umbrella term *embodied cognition*, despite differences between the research areas to which these terms refer. Embodied cognition links the symbolic and the perceptually founded as well as implicit personal aspects of word meaning. The observation that sensory and motor neural systems are directly engaged in language comprehension (Barsalou, 2008) upgraded and expanded the role of physical (including bodily) references to linguistic context, thereby pointing to the implicit experiential aspects of word meanings. Furthermore, the view that perception of the real world (for example, through visual input) affects word processing resulted in a situated approach to language processing wherein a wide spectrum of *context*-related features could influence language understanding: acoustic-phonetic, syntactic, semantic, pragmatic as well as affordances, social information and environment (Spivey and Hütte, 2016). Understanding how the combination of these different sources affect brain activity and behavior in language processes challenges research.

Interactions between perceptual/motor and semantic phenomena support embodied theories. Simply put, if linguistic representation is – at least partially – embodied, then perceptual and motor processes should influence how language is processed (and vice versa), leading to facilitation or interference. Validating and assessing the neural bases of such perceptuomotor-semantic interactions allows understanding the role the body and evironment play in language processes. This also represents a fundamental step towards biologically plausible theories of embodied semantics.

The aim of the paper is not to provide a comprehensive literature review in order to reiterate evidence for embodied views of language processing per se. Instead, we discuss a selection of available empirical findings in order to answer a set of specific theoretical and practical questions (see Figure 1 for an overview) concerning the brain signatures of embodied and situated language processing. We have paid special attention to critical issues related to the interpretation of novel insights and methodologies that examine the subtleties of perceptual-motor-semantic interactions, as well as future directions to be explored. The sections can be read as standalone sections, each ending with a special focus on future directions. In the final conclusion, we attempt to connect these selected puzzle pieces to the bigger picture. Please note that the authors of this consensus paper refrain from a militant all-or-nothing view on embodiment and support a moderate approach, leaving room for a flexible involvement of situated and embodied mechanisms in language processes.

**Figure 1:**
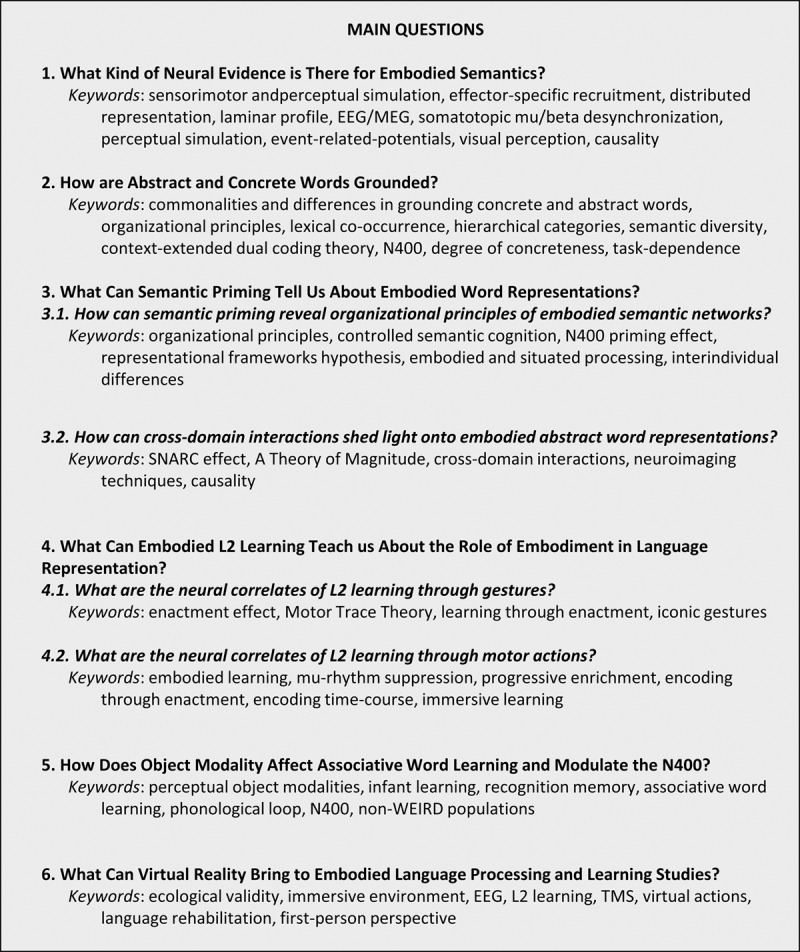
Overview of the addressed main questions.

## What Kind of Neural Evidence is There for Embodied Semantics?

Understanding the neural bases of embodied semantic processing requires the identification of the neurobiological markers that can be associated with sensorimotor simulation of linguistic meanings. This is a fundamental step towards developing theories of how embodied semantics is implemented at the level of neural circuits, as well as to make explicit predictions about the outcomes of experiments. In this section, we will review findings from different neuroimaging techniques that have contributed to the identification of distinct neural indices of sensorimotor simulation. For each methodological approach, we will address candidate mechanisms underlying the simulation of both the sensorimotor (e.g., recruitment of the motor system) and purely sensory (e.g., recruitment of the visual system) aspects of linguistic meanings.

Neuroanatomically, a number of findings supporting embodied meaning representations have primarily been based on a localization approach, aimed at estimating cortical structures activated during word comprehension outside core language areas. Such studies, for instance, showed that accessing the meaning of linguistic material is supported by the sensorimotor system. For example, a number of experiments showed that effector-specific cortical premotor and primary motor regions are recruited when individuals process words or sentences referring to actions carried out with different body parts (Aziz-Zadeh et al., 2006; Boulenger et al., 2009; Ge et al., 2018; Hauk et al., 2004; Kemmerer et al., 2008; Rüschemeyer et al., 2007; Tettamanti et al., 2005; but see Postle et al., 2008). Similarly, sensory brain regions were also found to activate during semantic processing. For example, processing words whose meaning is associated with auditory experiences (e.g., *telephone*) led to a larger brain activation in auditory areas compared to the processing of words denoting visual features (e.g., *moon*; Kiefer et al., 2008). Similarly, words referring to so-called multimodal concepts, that is, concepts that can be experienced through different modalities (e.g., *playing*) activate different modality-specific networks (i.e., visual and action-related; Van Dam et al., 2012).

These latter findings have led researchers to ask how semantic information distributed across different networks becomes integrated into coherent representations. Studies targeting this question have hypothesized the existence of convergence zones (Barsalou et al., 2003; Damasio et al., 2004) and one or several semantic hubs (Patterson et al., 2007), that is, central processing nodes that unify information from multiple networks into a coherent semantic representation. By means of a neurocomputational model it was shown that distributed circuits in hub areas can link motor and sensory semantic information (Tomasello et al., 2017); the authors suggested that semantic processing is distributed across category-general hubs as well as category-specific areas. Among others, the anterior temporal lobe (Lambon Ralph et al., 2010) and the parahippocampal gyrus (Epstein & Kanwisher 1998) have been proposed as candidate regions to bind and coordinate multiple representations. Yet, the exact contribution of these regions to semantic processing and the mechanisms that underpin it remain poorly understood.

Overall, the neuroimaging studies mentioned above (along with many others) provide some support for the claim that, at least in some contexts, conceptual language representations and sensorimotor processes are based on partially overlapping brain resources. Yet, to what extent the sensorimotor system supports meaning processing is still matter of debate. Moreover, the underlying computations performed by sensorimotor regions should still be at least to some degree distinct for language vs. sensorimotor processing. Thus, one future challenge for embodied semantics theories is to explain the dynamics that allow a given brain region to carry both conceptual and sensorimotor processing. Recent investigations of visual processing and imagery using laminar functional magnetic resonance imaging (fMRI), a neuroimaging tool capable of structural resolution at level of cortical layers, have shown that bottom-up sensory signals (visual input) and internally generated signals (e.g., visual imagery) are distributed across different laminar profiles (Lawrence et al., 2018). Extending these cases to language processing, sensorimotor simulation signals generated during word comprehension could be dissociated from sensory processing in terms of their laminar profile. Future studies using imaging techniques with laminar-resolved resolution are needed to assess how the same cortical circuit can process both sensory input and store word meanings at the same time. Similarly, comparing the processing of the same stimulus content presented in different modalities (i.e., linguistic and sensory) with time-resolved techniques may provide novel insights about the differences that characterize the semantic engagement from the perceptual-motor one (e.g., Giari et al., 2020).

Electroencephalography ([EEG]; Alemanno et al., 2012; Moreno et al., 2015; van Elk et al., 2010), magnetoencephalography ([MEG]; Niccolai et al., 2014) as well as electrocorticography (Canolty et al., 2007) studies have attempted to find electrophysiological markers of embodied semantics by looking at the *oscillatory properties* of the brain signal accompanying lexical-semantic language processing. Research concerning motor simulation has so far focused on embodied processes related to the visual or auditory presentation of single verbs or verbs embedded in sentences and associated with motor experience. Overall, results point to a specific role of oscillatory brain signals in certain frequency bands (i.e., alpha, beta, and closely related mu-rhythm) in implementing sensorimotor simulations. In particular, beta desynchronization (power suppression within the 13–30 Hz frequency range) was suggested as a candidate mechanism supporting the reactivation of motor experience during semantic processing, given its well-known role in preparation and execution of movements (Babiloni et al., 2002; Doyle et al., 2005; Koelewijn et al., 2008; Pfurtscheller & Lopes da Silva, 1999) as well as in isometric contraction of different body muscles (Tecchio et al., 2008). This has encouraged studies to investigate beta oscillations in the context of linguistic action processing in order to determine whether this activity pattern may constitute a neurophysiological marker of embodiment processes in other sensory modalities as well (e.g., auditory, see Niccolai et al., 2020). Results showed that loud actions induced significantly stronger beta power suppression compared to quiet actions in the left auditory cortex.

Along with beta suppression, the suppression of mu (8–13 Hz) rhythm, found over the sensorimotor cortex, is thought to reflect neural activity in the motor and premotor cortex and be associated with performing and observing movement (Caetano et al., 2007; Pfurtscheller & Lopes da Silva, 1999), motor imagery (Matsumoto et al., 2010), and, importantly, action language processing (Fargier et al., 2012). For instance, mu suppression has been observed during the processing of action-related sentences, during the retrieval of lexical-semantic information (van Elk et al. 2010), and in priming tasks using action words in first and second languages (Vukovic & Shtyrov, 2014). Somatotopic mu suppression has been observed while participants read single verbs related to the body (Niccolai et al., 2014); crucially, action language appears to produce greater mu suppression compared to abstract language (Alemanno et al., 2012; Moreno et al., 2015). However, a consensus has yet to be reached regarding the functional interpretation of mu-band activity suppression. Whereas some previous studies associated it with motor-cortex activity, the mu frequency band (8–13 Hz) overlaps with the alpha frequency band (8–12 Hz). While some authors claim that the two rhythms may be confounded (Hobson & Bishop, 2016), others argue that mu frequency reflects the functional state of the motor and premotor cortex and can be dissociated from occipital alpha by its fronto-central topography (Moreno et al., 2015) and motor-cortex localization of its sources (Vukovic & Shtyrov, 2014).

Concerning the generation of *perceptual* (rather than *motor*) simulation, a candidate mechanism to implement the simulation of perceptually based concepts (e.g., *red* or *triangle*) might be reflected by alpha-band oscillations. In visual perception, increases of alpha synchronization in occipital regions have been largely associated with top-down object-knowledge activation and maintenance during attention, prediction, and working memory tasks (Worden et al., 2000; Snyder and Foxe, 2010; Mayer et al., 2015; Jensen et al., 2002). Alongside its well-known role as inhibitory filter, current models of alpha oscillations posit that enhancement of neural alpha synchronization might reflect selective amplification of neural representations of object categories in task-relevant sensory regions (Palva & Palva, 2007; Klimesch, 2012; van Kerkoerle et al., 2014; Mo et al., 2011). Under this account, neural alpha synchronization might reflect a candidate mechanism to also implement language-mediated perceptual simulations. Although enhancements of neural alpha synchronization have not been reported ubiquitously during word processing, they have been consistently found in studies that used paradigms in which sensory processes are involved in language comprehension (e.g., word-picture matching task, sentence-shape verification task). Such tasks are particularly suited to assess the neural underpinnings of embodied semantic processing, as they require participants to mentally simulate the perceptual content of a given word or utterance to detect or recognize its referent. For instance, EEG studies have shown that processing spoken and written language material can facilitate the recognition of object categories and ambiguous images (Samaha et al., 2018; Morucci et al., 2021). Crucially, this facilitation is associated with an increase in posterior alpha synchronization in the time interval between the cue and the target image. The amplitude of alpha oscillations also seems relevant to object recognition processes, as it correlates with reaction times and early event-related components such as the P1 (Samaha et al., 2018; Mayer et al., 2015). These findings provide some support for the fact that alpha oscillations might reflect a mechanism to carry language-generated object representations in visual regions, mirroring the computational role that alpha waves play in visual perception (Palva & Palva, 2007; Klimesch, 2012; van Kerkoerle et al., 2014; Mo et al., 2011).

Oscillatory signatures of situated/embodied linguistic processes have also been found for more complex functions, outside basic sensorimotor modalities. For instance, it has been shown that comprehension of spatially-related language recruits brain mechanisms involved in navigation and spatial cognition (Vukovic & Shtyrov, 2017). This study used EEG to estimate activity in alpha/mu and beta ranges in both a spatial navigation task and a language task, which involved comprehension of sentences related to perspective/reference-frame concepts, and found a shared network of distributed cortical generators activated for both tasks, which, furthermore, was specific to individual navigation preferences.

Apart from oscillatory activity, a plethora of electrophysiological studies investigated the time course and magnitude of linguistic embodiment by means of *event-related potentials (ERPs) or fields (ERFs)*, most notably focusing on sensorimotor areas (e.g., Dalla Volta et al., 2014; Hauk & Pulvermüller, 2004; Klepp et al., 2014). Neurophysiological studies point to sensorimotor effects occurring between 80 and 350 ms accompanying the processing of single verbs as well as of action words embedded in sentences (Boulenger et al., 2012; Pulvermuller et al., 2005; Shtyrov et al., 2014). Some neurophysiological investigations complement behavioral measures of verbal-motor interaction (for a review, see Fischer & Zwaan, 2008) as well as priming and interference effects (for a review, see Garcia & Ibanez, 2016). Action-related words, for example, induced larger mismatch negativity-like ERPs when presented in body-part-incongruent sound contexts (e.g., *kiss* in footstep sound context) than in body-part-congruent contexts (e.g., *kick* in footstep sound context; Grisoni et al., 2016). In another study, finger button presses prior to the presentation of an arm-related word (e.g., *stir*) resulted in reduced brain activity in the hand knob compared to the incongruent conditions (e.g., *jump*), the related latency of 150 ms suggesting early semantic information retrieval (Mollo et al., 2016). Overall results from EEG and MEG show early and ultra-early effects of verb processing on sensorimotor areas as well as modulatory effects of motor priming or interference on cortical motor activation related to word processing. Furthermore, such effects have been documented even when the participants’ attention was diverted away from the linguistic input, suggesting a large degree of automaticity of sensorimotor involvement in language processing (Grisoni et al., 2016; Shtyrov et al., 2004, 2014). Still, behavioral effects of congruency between body-related action verbs (hand, foot) and body effector used for responses were shown to be larger when lexical decision or physical judgments tasks were performed, thus pointing to a role of attention and depth of processing in embodiment (Miller & Kaup, 2020). Also, semantic motor priming was shown to be effective only when a semantic task was required and not when semantic processing could be ignored (Klepp et al., 2017).

Potential ERP correlates of sensory simulation during language processing can also be found when examining the effects of language on *visual perception*. A critical question concerning language-perception interactions is whether language affects visual processing at early stages (i.e., by modulating activity in early sensory regions) or at later semantic or decision-making levels. The rationale of these studies is that, if words or utterances activate sensory representations of their referent objects in early sensory regions, then such representations should modulate early ERPs such as the P1 or N1 – putatively considered an electrophysiological index of low-level visual processes (Spehlmann, 1965). If, however, language only activates amodal representations, then no early sensory modulations should be found. Instead, later ERP effects might be found, such as the N400, typically associated with higher levels of object recognition. Studies using word-picture matching tasks showed that single words can bias visual processing of object categories as early as 100 ms from the onset of the image, with such modulations arising primarily in occipital regions (Boutonnet & Lupyan, 2015; Noorman et al., 2018). Similarly, processing words referring to facial body parts (e.g., nose) increases the amplitude of the N170 in the face-processing system to a larger extent than other body parts (e.g., hand). Interestingly, a machine-learning classifier trained to discriminate between facial body and non-facial body parts achieved better performance when trained on data from the face-processing system in the first 200 ms after word onset, compared to data based on the multimodal-network after 250 ms (Garcia et al., 2020). These findings suggest that at least some categories of content words activate content-specific visual representations in early sensory regions. Similar effects have also been reported when sentences precede visual object category presentation. For instance, hearing sentences about faces influences face processing by modulating the N170 component (Landau et al., 2010). Similarly, Hirschfeld et al. (2011) showed that ERP responses generated by incongruent sentence-picture pairs differed from those generated by congruent pairs around 170 ms after picture onset. Interestingly, studies using interference paradigms showed that visual noise disrupts the processing advantage for congruent word-picture pairs (Edmiston & Lupyan, 2017), but not for congruent sentence-picture pairs, which are instead affected by semantic noise (Ostarek et al., 2019). This finding suggests that while the processing of single words can lead to the activation of visual information, larger language units (e.g., sentences) might rely more on amodal knowledge representations. This is in line with models in which language comprehension engages two complementary systems, a perceptual and an abstract one (Mahon & Caramazza, 2008; Hirschfeld, 2011; Bi, 2021).

The functional relevance of modality-specific activation for the understanding of linguistic meanings remains a controversial issue. Theoretical models provide different views on the extent to which sensorimotor simulations contribute to semantic comprehension, with some theories claiming that simulations are necessary for language comprehension (Pulvermüller, 1999), others suggesting that they are context-dependent (Barsalou, 1999), and others limiting their functional role (Mahon & Caramazza, 2008). Causal paradigms provide some appealing methods to address this problem (See Ostarek & Bottini, 2021 for a review). These paradigms include studies on sensory and motor impaired individuals (e.g., blind or deaf individuals), lesion studies, and stimulation studies.

Studies on individuals with sensory and motor impairments provide a critical test for embodied semantics. The logic of these studies is straigthforward: if understanding the meaning of motor- (e.g., *kick*) or vision-related language (e.g., *sky*) requires the recruitment of motor and visual simulations respectively, then individuals with impaired motor or visual abilities should have a deficit in processing this type of language material. Evidence from these paradigms, however, is intermixed. For instance, processing action verbs activates the left middle temporal gyrus in both sighted and congenitally blind individuals (Bedny, Caramazza, Pascual-Leone, & Saxe, 2012), suggesting that the lack of visual experience does not impair semantic processing. Specifically, these findings have been taken as evidence for the fact that brain regions putatively activated during the processing of action verbs may represent amodal representations of verb meanings instead of visual-motion simulations. On the other hand, there is some evidence that specific dimensions of meaning, such as color concepts (e.g., “blue”), may be represented in different regions in sighted and blind people. For instance, while processing color words activates regions known to represent low-level visual features (e.g., the posterior occipital cortex) in sighted people, individuals born blind seem to rely more on temporal regions, that is, regions usually associated with lexical-semantic processing (Bottini et al., 2020). This latter result suggests that sensory simulations may be recruited during the processing of some specific meanings, but that they are not necessary, or always available, during semantic processing. It must also be noticed that previous studies showing similar activation profiles in sighted and early blind people processing language material do not necessarily indicate that sensorimotor simulations are not recruited during semantic processing. Indeed, there is evidence that occipital regions reorganize for other sensory computations in the absence of vision (e.g., auditory, gustatory). Therefore, a similar pattern of activation over occipital regions may reflect different simulatory mechanisms in the sighted and blinds (Ostarek & Bottini, 2021).

Another approach to test the causality of sensorimotor simulation to semantic processing relies on lesion studies. For instance, some studies on cortical motor lesions show a lack of impairment in action word processing that embodied theories instead would postulate (Arevalo et al., 2012; Weiss et al., 2016), while others suggest action-language specific impairments when the motor system is damaged (Bak & Chandran, 2012). The effect of pathological changes in the motor system such as in Parkinson’s disease can contribute to the investigation of the causality issue: we refer the reader to section 5 of the paper by Ibáñez et al. in this journal issue for an in-depth discussion.

In healthy populations this has been tackled by applying neuromodulation techniques such as transcranial magnetic stimulation (TMS) and transcranial electrical stimulation (tES), and targeting the hand and foot motor areas during the processing of effector-specific action verbs and sentences. Results show that stimulation modulated reaction times and/or cortical excitability, although some inconsistencies remain as for the direction of the modulation in the sense of cortical motor inhibition vs. facilitation (Buccino et al., 2005; Gianelli & Dalla Volta, 2014; Lo Gerfo et al., 2008; Niccolai et al., 2017; Oliveri et al., 2004; Papeo et al., 2009; Pulvermuller et al., 2005; Repetto et al., 2013; Scorolli et al., 2012; Weiss et al., 2016; Willems et al., 2011). Such inconsistencies may stem from diverging stimulation settings (e.g., supra- vs sub-threshold, single-pulse vs. repetitive) and different linguistic tasks involved across different studies. For instance, it has been showed that, whereas TMS of hand motor areas does not affect hand-related word processing in a lexical decision task, a deeper level of processing prompted by semantic judgment task is compromised by TMS in a meaning-specific fashion (Vukovic et al., 2017), as is action-word acquisition (Vukovic & Shtyrov, 2019). Further, performance in a semantic discrimination task seems to affect the effect of tES on priming (Niccolai et al., 2017). Thus, although overall results point to somatotopically organized engagement of cortical motor areas in the understanding of written and spoken action, the issue of causality remains debated and is in need of further studies that should more systematically explore different modalities, stimulation regimes and linguistic contexts.

### Future directions

Although the reviewed markers point to an involvement of sensorimotor systems in language processing and provide clues as to the location and timing of their engagement, they are still limited in some of their details. For instance, the sign of neural interactions subserving simulation mechanisms is still poorly understood, as fMRI and EEG findings are usually not informative about whether neural activity reflects inhibitory or facilitatory processes, or a combination of both. Similarly, interpretations of TMS findings remain somewhat ambiguous because experimental conditions are often contrasted with each other, without the consideration of an appropriate baseline. Besides these methodological considerations, it remains to be determined how exactly sensorimotor areas can engage in perceptual/motor as well as linguistic information processing in functionally specific ways, and what mechanisms enable this functional switch from perception/execution to embodied semantic processing. Further, it has been shown that not only verbs and nouns can modulate sensorimotor activation. For example, the advantage shown by the 1^st^ vs. 3^rd^-person perspective in activating specific brain areas such as the posterior cingulate area, the right superior temporal sulcus, the pre/motor and the somatosensory area (Tomasino et al., 2007; Ruby & Decety 2003; Niccolai et al. 2021; Wagner et al. 2014) points to a role of the situational context in embodied linguistic processing (see also section 4 of Ibáñez et al., in this journal issue). Beyond verbs (for a review, see Fischer & Zwaan, 2008), nouns (Carota et al., 2012), pronouns, and adjectives (Gough et al., 2013), adverbs can also modulate sensorimotor activation (Sieksmeyer et al., 2021). While replications of these findings is strongly needed, it would be important to elucidate whether these syntactic elements contribute to the shift from embodied to more situated semantics.

## How are Abstract and Concrete Words Grounded?

Language is a powerful vehicle for the expression of knowledge: by mapping mental representations of objects, actions, and facts of the world onto word meanings, it allows individuals to share thoughts, experiences, and feelings, in order to be understood by others. For example, people can talk about things they can experience directly through their senses, such as *dog* and *pizza*, but they can also talk about more abstract entities, such as, *intelligence* and *justice*, which constitute around 70% of the words individuals use daily (Bolognesi & Steen, 2018; Recchia & Jones, 2012). These latter concepts pose a problem for theories that ground knowledge in sensorimotor systems (e.g., embodied or grounded cognition theories). Indeed, how can sensorimotor activation systems represent words that do not appear to be grounded in these systems? Within the general aim of this consensus paper, this section addresses this question from various perspectives by examining studies comparing abstract and concrete word processing, in order to provide an overview of current approaches to the possible commonalities and differences in the grounding of these words and to suggest future directions in this strand of research. The *conceptual metaphor theory* addressed this question by observing that in language, concrete words are often used as a metaphor to talk about abstract concepts (Lakoff, 1980; Pecher, 2018). A body of behavioral data supported this theory, showing that people activate some sort of spatio-motor representations when they talk and think about abstract concetps, consistent with embodied semantics. On the other hand, neuroimaging studies observed mixed results. For instance, when comparing metaphorical sentences including action words and action-related literal sentences, while Aziz-Zadeh et al. (2006) failed to report motor activation for the idiomatic sentences, Boulenger et al. (2009) found this activation. Yet, it is unlikely that all abstract concepts can be grasped thanks to their metaphorical relations with concrete concepts (Borghi & Zarcone, 2016). An alternative idea is that, compared to concrete words, abstract ones have more situation-dependent properties (Wilson-Mendenhall et al., 2011). Providing situational information often facilitates the processing of abstract words (Murphy & Wisniewski, 1989; Schwanenflugel & Shoben, 1983; Wattenmaker & Shoben, 1987), suggesting that situations are important for understanding these types of words.

In the literature, the property that determines whether a word is concrete or abstract has been termed *concreteness*. By definition, concreteness indicates the degree to which a word refers to an entity that can be perceived through the external senses. This dimension is usually assessed by participants on Likert scales (Brysbaert et al., 2014; Montefinese et al., 2014), in which concrete words lie on one side of the scale, referring to single, bounded, identifiable referents that can be perceived through the senses (Borghi et al., 2017). Abstract words lie on the opposite side of the scale and lack clearly perceivable referents with the affective words more strongly reliant on interoception (i.e., sensations inside the body; Connell et al., 2018; Montefinese et al., 2020). Indeed, compared to concrete words, abstract ones are acquired later and mostly through language and social interaction rather than physical sensorimotor experience (see Borghi et al., 2019; Dove et al., 2020). Concrete words are more imageable (Paivio, 1990) and have greater availability of contextual information (Schwanenflugel et al., 1992). Different organizational principles have been argued to govern semantic representations of concrete and abstract words (Montefinese, 2019). Indeed, concrete words are predominantly organized by similarity in the sensorimotor experience (Montefinese et al., 2015; 2018a), as has been inferred from asking participants to list properties of the concept which a word refers to (Buchanan et al., 2019; Montefinese et al., 2013; Montefinese & Vinson, 2015). Abstract words, in turn, are mostly organized by associative relations, which indicate the extent to which words are bound in the mind (as inferred by a free word association task; Crutch & Warrington, 2005; Montefinese et al., 2018b). It has also been proposed that abstract word representation could be based more on semantic similarity arising through patterns of words’ co-occurrence in linguistic contexts and syntactic information, based on the idea that words with similar meanings are mostly used in similar linguistic contexts (Vigliocco et al., 2013). However, some studies in the literature have questioned the latter claim by showing that lexical co-occurrence-based models either predict the processing of concrete and abstract words similarly (Rotaru et al., 2018) or account better for concrete than abstract words (Hill et al., 2013; Montefinese et al., 2021; see also the next section).

Concrete words can also be inscribed into definite domains, such as natural objects vs. artifacts, plants vs. animals, etc., and they are organized into hierarchical categories (animals/dogs/terriers/), while abstract words are considerably more variable with regard to their semantic content and they cannot be contrasted as a uniform category with concrete words (Borghi et al., 2017; Montefinese et al., 2015; but see Troche et al., 2014). Moreover, participants agree more when they produce properties and associations for concrete words like *dog* and *pizza* compared with abstract words like *intelligence* and *justice* (De Mornay Davies & Funnell, 2000; Tyler et al., 2002). This could be due to abstract words generally having a greater number of possible meanings (i.e., polysemy or *semantic diversity*) compared to concrete words (Hoffman et al., 2013). This ontological distinction is reflected in their different processing and representation in the human brain. Indeed, a mounting body of evidence from functional neuroimaging studies supports the idea that the left and right cerebral hemispheres differ in their processing of concrete and abstract words (e.g., Binder et al. 2005; Dhond et al., 2007; Pexman et al. 2007; Sabsevitz et al. 2005). The general consensus from these studies is that two main systems underlie the processing of these types of words, with abstract words relying on the verbal-language system and concrete words relying on the imagery and perceptual systems (Wang et al., 2010) as previously suggested by the seminal work of Paivio, who conceptualized this in his dual coding theory (Paivio, 1990, 1991; see below).

Moreover, behavioral research over several decades has found that concrete words are processed more quickly and accurately than abstract words (for a review, see Huang & Federmeier, 2015). This processing advantage is labeled *concreteness effect* and has been observed in a variety of tasks. For example, as compared to abstract ones, concrete words are responded to more quickly in lexical decision tasks (Bleasdale, 1987; Kroll & Merves, 1986; but see also Kousta et al., 2011), are easier to encode and retrieve (Romani et al., 2008; Miller & Roodenrys, 2009), are easier to make associations with (de Groot, 1989), and are more thoroughly described in definition tasks (Sadoski et al., 1997). Although it has recently been suggested that some of these effects may be driven by surface word properties and their acquisition backgrounds rather than semantics per se (Kurmakaeva et al., 2021) and some studies have even documented the reverse of concreteness effect when surface properties are matched (Mkrtychian et al, 2021), the evidence in favor of general concreteness word advantage remains ubiquitous.

Two main competing theories have been proposed to explain the concreteness effect. As mentioned above, the *dual coding account* (Paivio, 1990) claims the existence of two processing systems: a verbal symbolic system responsible for the representation and processing of linguistic information, and an imagery system for the nonverbal information. This theory argues that abstract words are only represented through a verbal code, while concrete words are represented using both a verbal and non-verbal code, resulting in an additive effect and processing advantage for concrete words over abstract words (Paivio, 1990). Thus, similarly to the amodal theories (Mahon & Caramazza, 2008), Paivio‘s theory neglects the grounding of abstract meaning and claims that only a symbolic amodal system plays a role in the processing of abstract concepts. Within the dual coding framework, a hybrid view combining an embodied approach with a distributional one seems promising (Andrews, Frank & Vigliocco, 2014; Montefinese, 2019). Indeed, distributional models based on linguistic data appear to be appropriate for describing abstract concepts (Louwerse, 2011).

In contrast to the dual coding account, the *context-availability model* (Schwanenflugel, 1992) pays no attention to the special status of the perceptual information, rather it claims that abstract and concrete words are represented in a single symbolic system. This theory assumes that comprehension relies on context supplied by either the preceding discourse or the comprehender’s own semantic knowledge. Concrete words are more closely associated with the relevant contextual knowledge in semantic representation because they have stronger or more extensive connections to this stored knowledge compared with abstract words. Thus, the poorer performance for abstract words is due to the relative unavailability of associated contextual information in semantic representation for these words (Schwanenflugel, 1992). Abstract words may be associated with a wider variety of situations than concrete concepts (Galbraith & Underwood, 1973) given their greater number of meanings, as mentioned above (Hoffman et al., 2013). As a result of greater interference between competing situations, retrieving a single one may be more difficult for an abstract word than for a concrete one.

Unlike laboratory studies where words are presented in isolation, when people process abstract words in the real world, a relevant situation is already in place and this could facilitate the processing of abstract words by resolving the interference between competing situations. Indeed, some studies observed that differences between concrete and abstract words are reduced when abstract words have sufficient contextual support (Schwanenflugel, 1991; 1992; see also the next section). In sum, in both the dual coding and the context availability accounts, concrete semantic representations are posited to be richer than abstract semantic representations. Moreover, they both overlook the contribution of the semantic grounding and embodiment in the organization of abstract representations.

EEG and, in particular, the ERP technique is particularly suitable to study functional differences in the processing of abstract and concrete words due to its high temporal resolution. Thus, ERP studies may unveil the time-course of multiple processes that are summated in behavioral measures (Huang & Federmeier, 2015). The ERP literature on the concreteness effect appears generally consistent across a wide variety of cognitive tasks, such as, imageability ratings, lexical decisions, go-no tasks, congruency and semantic judgments, implicit and explicit memory tasks (e.g., Adorni & Proverbio, 2012; Barber et al., 2013; Kanske & Kotz, 2007; Lee & Federmeier, 2008; Swaab et al., 2002; West & Holcomb, 2000). Overall, ERP studies show an N400 component (a negative-going waveform in centro-parietal electrode sites peaking around 400 ms post-stimulus) generally associated with the processing of meaning and in particular with semantic access (for a comprehensive review, see Kutas & Federmeier, 2011). ERP studies on the concreteness effect showed a larger N400-like component for concrete words compared with the abstract ones generally spanning from 300 to 500 ms after the onset of the target stimulus, often extended to the frontal sites. In accordance with the context availability theory, this pattern of results could be driven by the more availability of contextual information for concrete words. Another interpretation posits that concrete words evoke more activity in semantic representation eliciting a larger N400 (Barber et al., 2013) because of their richer semantic networks (in terms of multimodal features), supporting the claim of a strong grounding in the sensorimotor experience of concrete words. Indeed, in line with the idea that the N400 reflects the level of meaning activation (Molinaro, Conrad, Barber & Carreiras, 2010), the authors proposed that when words are presented out of context, compared to the concrete concepts, the abstract ones would activate superficial connections with their associate words, resulting in a shallower activation process. This is consistent with the fact that when the words are presented with a context (like in the priming studies) the N400 concreteness effect disappears (as discussed in the following section).

A concreteness effect has also been found in ERP amplitudes as early as 150 ms (Mkrtychian et al., 2021) and a at later time of the sustained frontal N700 (typically spanning between 300 and 900 ms), consistent with the dual-coding account, which attributes the concreteness effect to the availability of sensorimotor imagery primarily involved in concrete word processing (Huang & Federmeier, 2015). This latter pattern of results provides once again evidence for a semantic grounding of the concrete words. Holcomb et al. (1999) speak in favor of a *context-extended dual coding theory*, which integrates (but it also differentiates from) the context availablity and dual coding theories at the neurophysiological level. Upon closer inspection, while the context availability would posit larger context effects for abstract words, the context-extend dual-coding theory would predict similar effects for abstract and concrete concepts within the verbal system. Moreover, it would posit larger contextual effects for concrete than abstract words in the imagery system. This account is supported by many neuroimaging studies (e.g., Bechtold et al., 2022; West & Holcomb, 2000; Binder et al., 2005; Zhang et al., 2006), still generally supporting the idea that the neural processing of abstract and concrete words is different.

### Future directions

In light of these considerations, context availability and dual coding theories do not exhaust the differences between abstract and concrete words. However, it is possible that taking only grounded information into account might not suffice to capture the semantic representation of abstract words. Indeed, rating and property generation studies suggested that word representation is characterized as a differential conceptual feature composition (Barsalou & Wiemer-Hastings, 2005; Binder et al., 2016; Troche et al., 2014). In order to better account for the representation of abstract concepts, refined grounded cognition approaches posited the relation between modality-specific brain systems and abstract meaning (Kiefer & Harpaintner, 2020). These accounts were supported by a limited number of fMRI studies, observing that the processing of abstract meaning, like mental states, number and emotion, was associated with an increased activity in the motor brain areas (Dreyer & Pulvermüller, 2018; Tschentscher, Hauk, Fischer, & Pulvermüller, 2012; Moseley, Carota, Hauk, Mohr, & Pulvermüller, 2012). These approaches emphasize for example, the importance of linguistic/verbal (Barsalou, Santos, Simmons & Wilson-Mendenhall, 2008; Borghi & Binfolski, 2014), social (Barsalou & Wiemer-Hastings, 2005; Borghi & Binfolski, 2014), and affective (Kousta et al., 2011; Lenci et al., 2018) information in abstract representation, resonating partially with the dual coding account. Unfortunately, most of the ERP literature tested classical theories (i.e., the dual coding and context availability theories) on semantic representations of abstract and concrete concepts (for an exception see Harpaintner et al., 2020), consequently overlooking the contribution of the embodied ground of experience to their semantic representation.

Moreover, all ERP studies mentioned above manipulated concreteness factorially, thus oversimplifying the complexity and variability of this variable. However, a critical aspect of the concreteness effect is the non-dichotomous nature of concreteness: even if concrete and abstract words differ, they do not represent distinct classes and no clear-cut boundary can be drawn between them. Instead, a word’s concreteness should be seen as lying along an concreteness continuum and it can be approximated by taking the mean value of participants’ ratings (Borghi et al., 2017).

Myachykov and Fischer (2019) proposed a hierarchical and componential view of concreteness, in which abstract and concrete word representations reflect the experiences in the physical world (*grounded* component), the bodily constraints (*embodied* component), and the changeable context in which the words are used (*situated* component). Therefore, words would be positioned along a multidimensional concreteness continuum depending on the relative weight of these components, where the same word may vary and be both abstract or concrete depending on the specific context and different dimensions considered. For this reason, in order to better track the time course of the concreteness effect in word processing, it would be particularly important to investigate the continuous effect of this variable on participants’ behavioral and ERP responses. It must be also noted that inconsistent results across ERP studies may be due to differences in confounding variables for abstract and concrete words, such as word frequency and to the different types of task, context, and encoding (Huang et al., 2010; Pexman et al., 2007). Controlling for these potentially confounding factors is thus crucial in the research assessing the processing of abstract and concrete words, especially because semantic representation

“is a dynamic and transient entity, constructed on the fly according to the constraints of task demands, context, available resources, and processing goals, and includes perceptual, motor, affective, situational, and linguistic (i.e., label) information to greater or lesser degrees” (Connell, 2019: 1310).

Unfortunately, to date, only few ERP studies have tackled this issue. These studies showed that the presence/size of the concreteness effect is sensitive to the type of the task (e.g., Gullick et al., 2013; Welcome et al., 2011; West & Holcomb, 2000; Xiao et al., 2012). In particular, a larger concreteness effect has been observed in tasks emphasizing imagery and the explicit processing of semantic properties compared to tasks emphasizing the processing of surface properties of words. However, tasks emphasizing the processing of affective information could invert the concreteness effect in favor of abstract words, highlighting the embodied grounding of abstract representation. Importantly, assuming a situated grounding also for abstract words, putting words in context could help to disambiguate among the multiple meanings which abstract words refer to (see next section for more details).

## What Can Semantic Priming Tell Us About Embodied Word Representations?

In real life, we rarely process words in isolation. Thus, to draw ecologically valid conclusions about the mechanisms of embodied language processing in the human brain, it is important to look at how word processing varies according to the (linguistic) context in which it occurs. One powerful tool to embed words in context and still disentangle mechanisms involved in word vs. context processing is *semantic priming*. In semantic priming, a semantically congruent *prime* facilitates the processing of the following *target* word, which leads to shorter reaction times or higher accuracy, with the opposite pattern for incongruent prime-target combinations (Meyer & Schvaneveldt, 1971; for a review see Neely, 1991). Possible neural mechanisms underlying priming effects include forward- and backward-directed mechanisms. Forward-directed mechanisms have been discussed to include (automatic) spreading activation in the semantic network (Collins & Loftus, 1975; Kiefer, 2002) and predictions (Delaney-Busch et al., 2019; Lau et al., 2016) or controlled expectancy generation (Aurnhammer et al., 2021; Frade et al., 2022). Backward-directed mechanisms include integration and re-evaluation/prediction-error adaptation (Jack et al., 2019; Steinhauer et al., 2017). These processes can act within the semantic network connecting the prime and target word as well as across domains. Thus, measuring behavioral or neural consequences of semantic and cross-domain priming allows inferences about the organizational principles of embodied word representations.

To find out what semantic priming can tell us about embodied word representations, we focus on two lines of research in the following. The first line directly takes up the differences between concrete and abstract words presented in the section above and focuses on what concrete vs. abstract word priming effects driven by similarity- and association based relations can reveal about the organizational principles of lexico-semantic word representations. The second line focuses on what cross-domain priming within the abstract domain of numerical cognition can reveal about embodied perceptual information in abstract word representations. Finally, we will provide future directions on how to exploit the potential of semantic priming paradigms in embodied and situated language processing research.

### How can semantic priming reveal organizational principles of embodied semantic networks?

The differences in semantic representation of concrete and abstract words described in the section above should modulate the influence a prime exerts on concrete and abstract word processing, measurable in the size of priming effects. The dual-coding theory (Paivio, 1990, 1991) and context-availability model (Schwanenflugel & Shoben, 1983; Schwanenflugel & Stowe, 1989) would predict differences in the magnitude of priming effects based on qualitative and quantitative differences in the embodied information underlying concrete and abstract word representations. Both theoretical approaches have been integrated into the *controlled semantic cognition* framework (Hoffman et al., 2018; Lambon Ralph et al., 2017) assuming an interplay of concreteness- and context-driven semantic processes. In ERP studies, interactions of concreteness and prime congruency on the N400 manifested in different ways: for instance, embedding concrete and abstract words in a congruent sentence was shown to cancel out the anterior, lateralized N400 concreteness effect (Holcomb et al., 1999), whereas a congruent single-word prime reduced the N400 earlier for abstract than concrete words (Wirth et al., 2008) or contextual sentences reduced the N400 in response to concrete words thereby cancelling out the concreteness effect (Bechtold et al., 2022). Other N400 studies manipulating concreteness and prime congruency found only independent effects but no interaction of the two factors (Grieder et al., 2012; Swaab et al., 2002). None of these results mirror the stronger contextual modulation of reaction times elicited by abstract words (see, e.g., Bechtold et al., 2021; Bechtold et al., 2022), highlighting a dissociation between the behavioral vs. electrophysiological level. Barsalou (2020) pointed out that cognitive mechanisms like priming, even if the underlying processes occur automatically, are situated and context-dependent and their replicability relies on numerous factors, restricting their generalizability.

Additionally, the heterogeneity in previous findings might be due to imprecisions and/or diversity in how the studies defined the *semantic* in semantic priming. Different semantic relations among words, such as similarity and association, seem to elicit different priming effects on behavioral, electrophysiological, and neural activation measures (Sachs, Weis, Krings, et al., 2008; Sachs, Weis, Zellagui, et al., 2008; Sachs et al., 2011). The *representational frameworks hypothesis* (Crutch & Warrington, 2005) states that the experience with concrete and abstract concepts embeds them in different representational frameworks in semantic memory, resulting in a differential reliance on specific semantic relations. Based on sensorimotor experience, concrete concepts are thought to be interrelated via *perceptual similarity*. For example, a *dog*, a *wolf*, and a *coyote* all share the perceptual appearance of a furry, four-legged canine (i.e., show a considerable feature overlap; Kellenbach et al., 2000), which interconnects their representations and makes room for perceptual semantic priming effects. Abstract concepts on the other hand are mostly experienced in situational or linguistic contexts (Hill et al., 2014), where words like *intelligence* co-occur with words like *test* and *knowledge*. For abstract concepts, *thematic associations* (e.g., derived from free word association norms), which are partly based on but not equal to *lexical co-occurrences* (Montefinese et al., 2018b; Montefinese et al., 2021), are thus an important factor determining their interrelations. The differential frameworks hypothesis was based on behavioral evidence from odd-one-out tasks (Crutch, Connell, & Warrington, 2009; Crutch & Jackson, 2011), in which participants were presented with a row of 3–5 words, which were either related by association or similarity, except for the one odd word. These studies could show with patients as well as healthy participants that concrete odds pop out more easily from similar words and abstract odds from associated words, facilitating their identification. At the behavioral level, the differential reliance of abstract words on association was replicated using a visual world paradigm (Dunabeitia et al., 2009), but neither single word priming (Ferre et al., 2015; Montefinese et al., 2018b) nor translation tasks (Geng & Schnur, 2015; Zhang et al., 2013) found evidence in support of the differential frameworks hypothesis.

At the electrophysiological level, it has been shown that functional similarity elicited priming effects on semantic integration (as reflected by the N400) whereas thematic association elicited priming effects on earlier word recognition (N1) and attention (P3; Wamain et al., 2015). In contrast, other ERP studies found only association- but not similarity-driven (Savic et al., 2017) or no differences between association- and similarity-driven N400 priming effects (Chen et al., 2013; Khateb et al., 2003). Based on assumptions of the differential frameworks hypothesis, this heterogeneity in findings is hardly surprising as these studies did not include the factor of concreteness. Further, the effectiveness of forward-directed mechanisms in priming should be directly influenced by the prime’s semantic diversity, as it determines how stable the semantic representation of the prime (Hoffman et al., 2013) and thus its relation to the target can be. Further, it has been shown that there are interindividual (Mirman & Garziano, 2012) as well as cultural (Uskul et al., 2008) differences in the preference to process either taxonomic/similarity-based or thematic/associative relations. These variables possibly strongly modulate N400 priming effects of associative and similarity-based relations on concrete vs. abstract words.

#### Future Directions

To conclude, primed word processing allows us to investigate embodied and situated semantics depending on the prime’s and target word’s psycholinguistic features as well as their interconnections within the embedding semantic network. Therefore, semantic priming is at the cross-roads of embodied and situated language processes, which is an opportunity and a challenge at the same time. As the word’s representational substrates as well as the embedding representational framework are strongly experientially grounded, it is crucial that future research takes into account experience-driven interindividual differences (Barsalou, 2020; see also Ibáñez et al. in this issue) in priming effects when designing experiments and analyzing their results. Further, it is important to acknowledge the multidimensionality of different kinds of semantic relations (e.g., taxonomic, perceptual-similarity, functional similarity, antonyms, valence, lexical, or thematic co-occurrence), which have often been summarized under the umbrella term semantic priming. Measuring ERPs, including, importantly, the N400, is still a powerful method to assess differential embodied priming effects at the neural level; however, future research has to approach its functional interpretation with caution, as it is sensitive to manifold forward- and backward-directed processes in word processing.

### How can cross-domain interactions shed light onto embodied abstract word representations?

Previous research on cross-domain priming focused largely on the concrete domain and specifically on action semantics (see Willems & Hagoort for a review). One prominent example is the well-known *action sentence compatibility effect* (Glenberg & Kaschak, 2002; Morey et al., 2021; see also section *Sentences* in Körner et al. in this issue). A recent theoretical approach links cross-domain action language priming to the activation of common representations by language and action observation, which they called *semantic resonance* (Bidet-Ildei et al., 2020). Given the origins of cross-domain priming, it is not surprising that most studies investigating abstract word priming are based on the assumption that sensorimotor experience plays an important role in semantic representations of abstract words as well (Barsalou, 2009). It has been shown that processing abstract concepts such as emotionally valenced words (e.g., Meier and Robinson, 2004), time-related words (e.g., Gevers et al., 2003; Gevers et al., 2004) and numbers (e.g., Loetscher et al., 2010) is accompanied by activation of, e.g., the visual attention system and the motor system. These sensorimotor effects were supported by Spatial Numerical Association of Response Code effect (SNARC; Dehaene, Bossini, & Giraux, 1993), Spatial Temporal Associations of Response Code effect (STEARC; Ishihara et al., 2008), and other SNARC-like effects, e.g., those found for musical tones (Lidji et al., 2007) and physical size (Prpic et al., 2020). In these studies, representatives of European cultures reacted faster to relatively small numbers and past-related words when using their left hand (or left answer button) and for relatively large numbers and future-related words when using their right hand (or right answer button), which has been linked to both reading and finger counting directions. This phenomenon in abstract word processing might be considered as evidence for a crucial involvement of sensorimotor systems for processing of each single abstract domain (i.e., numbers and time; see also section *Word–Space Associations* in Körner et al. in this issue).

A relatively open question is whether interactions between abstract concepts from *different* domains rely on embodied principles when these concepts are co-activated in one experimental task. Recent priming studies already showed that interactions between such representations as *number* and *size* (Lindemann et al., 2007), or *number* and *weight* (Holmes & Lourenco, 2013) are accompanied by reaction time facilitation effect. Myachykov et al. (2017) suggested that such partially related representations might interact through their common neural architecture. According to *A Theory of Magnitude* (Walsh, 2003), such domains are supposed to be a part of a generalized magnitude system based on overlapping connections in the parietal cortex. Indeed, associations such as “1 is small and 9 is big” or “small things are light and big things are heavy” are widespread and intuitive.

A phenomenon which is somewhat opposite to partially related representations is cross-domain interactions between concepts that are not directly related to each other either semantically or associatively, e.g., *time* and *magnitude*; *emotionally-valenced words* and *numbers* etc. According to Myachykov et al. (2017), semantically unrelated representations might still interact through the attention system or working memory. In this sense, abstract concepts that rely on a different neural architecture (e.g., *emotions, time*) might become co-activated in the same experimental task as they correlate in their effect on attentional and working memory resources. Indeed, if the same attentional shifts (to the left/right side) are cаused by processing of both time-related words and numerical semantics, then we can assume an interaction between them through the activation of an online interface causing semantic priming effect. Although this area is still understudied, there are some studies describing semantic priming effects for interactions between abstract concepts and concrete words that are not related semantically, e.g., between *words with concrete spatial referents* and *numbers* (Lachmair et al., 2014a, 2014b).

To summarize, at least two potential mechanisms of cross-representational interactions are suggested: representational overlap for partially related representations (common neural substrate of representations in specific domains), and an online interface for relatively unrelated representations (domain-general cognitive mechanisms, such as attentional and working memory). Although empirical evidence for these theoretical suggestions is still in development, we can state that the abstract word priming is a powerful tool, which allows us to look deeper into mechanisms underlying embodied language processing.

#### Future directions

The nature of cross-domain priming is still understudied; therefore, potential mechanisms of cross-domain interactions remain hypothetical. Future research on the topic might benefit from development of accurate paradigms that can separate the two types of cross-domain interactions (overlap in representational content vs. general cognitive mechanisms). The clear division (if such division exists) may help to clarify the nature of concepts and understand the mechanisms of their occurrence. Second, most studies of embodied abstract word representations are based on behavioral (less often on eye-tracking) data, which generally does not allow to investigate temporal dynamics of abstract concept processing in the brain. The use of brain imaging techniques, especially with high temporal resolution (e.g., MEG, EEG) is required to address this issue. Finally, correlational relationships (coactivation of general systems of consciousness – e.g., the attention system when processing concrete or abstract concepts) does not necessarily mean causality or imply that without this coactivation their processing would be impaired (see e.g., Mahon & Caramazza, 2008). Therefore, the most fundamental future direction is testing the whether the sensorimotor effects are merely side effects or artifacts or whether they truly reflect the embodied nature of abstract concepts (see e.g., Fernandino et al., 2022), which could be tested using techniques able to address causality, such as non-invasive brain stimulation.

## What Can Embodied L2 Learning Teach us About the Role of Embodiment in Language Representation?

Understanding how we first associate concepts to words is crucial for grasping how we represent language. In line with the Hebbian theory of associative learning, the “correlational learning principle” claims that the co-occurrence of action-perception and meaning results in the common firing of neurons, forming “embodied referential semantic circuits” to support meaning representation (Pulvermüller, 2013). Indeed, studies on verbal memory in the native language (L1) have shown that self-performed actions, associated to word labels during encoding, boost memory performance and supports word learning, a phenomenon called *enactment effect* (Engelkamp & Zimmer, 1985). More recently, learning language with self-performed or self-generated action that is directly linked to the learned content has been referred to as “embodied learning”. In this sense, studies on second language learning (L2) can provide relevant insights concerning the role of embodiment in language processing. Indeed, neurocognitive studies have become increasingly interested in scrutinizing motor-semantic interactions from initial word encoding in L2 to better understand the role that embodiment may play in language learning (i.e., facilitation). As we will discuss in the following subsections, drawing links between associative language learning and sensorimotor processes has proven both fruitful and extremely complex, pointing to a promising avenue in studies examining embodied processes.

### What are the neural correlates of L2 learning through gestures?

According to the embodied language perspective, not only is our native language (L1) grounded in perceptual and motor information, but also L2 benefits from the interplay between the linguistic and the sensorimotor system (Vukovic et al, 2014; Monaco et al., 2019). Indeed, behavioral studies indicated that when words are encoded along with multisensory information, they are better remembered and more resistant to decay (De Nooijer et al., 2013; Macedonia, 2003; Macedonia & Knösche, 2011; Repetto et al., 2017). While this enrichment can be accomplished in different ways, i.e., adding pictures to words being learned, the literature (in line with the tradition in the field delineated in the previous sections of this paper) has paid special attention to the role of the motor system in L2 acquisition. In these studies, self-performed gestures are generally added to word labels during the learning phase, leading to the replication, in L2 learning settings, of the enactment effect initially discovered in L1 verbal learning (Engelkamp & Zimmer, 1985). One possible explanation for the enactment effect is provided by the *Motor Trace Theory* (Engelkamp, 2001). According to this theory, when a word is learned together with a gesture, the motor information becomes part of its semantic representation; thus, this motor trace contributes to the multimodal representation involved in item-specific processing and in turn improves memory performance. This hypothesis is supported by neuroimaging studies that have shed light on how L2 words encoded through gestures are represented in the brain. For example, Macedonia and Mueller (2016) explored the neural correlates of word learning via enactment. Participants underwent a word recognition task in the scanner after a 4-day enactment-based training phase during which they learned new words in association to either iconic gestures or semantically unrelated gestures. Iconic gestures are defined as

“gestures that have some physical resemblance to the meaning or idea that it stands for” (APA Dictionary, https://dictionary.apa.org/iconic-gesture, retrieved on march the 1st, 2022).

When contrasted against resting state, results showed that words learned in association with iconic gestures activated a complex cortical and subcortical neural network including the left premotor cortex (BA 6), primary motor cortex, putamen, substantia nigra, left and right caudate, and the bilateral cerebellum. The authors interpreted these findings as evidence of the reactivation of an experience-related network involved in enacted word encoding. In a similar experiment, Mayer et al. (2015) found that specialized visual and motor areas are involved in representing words only when they are learned with support of motor (e.g., gestures) and visual (e.g., pictures) cues. Using multivariate pattern classification technique, the authors were able to identify the superior temporal sulcus and the premotor cortex as core regions representing words trained with gestures, and the right anterior lateral occipital complex as the core region related to the representation of words encoded with pictures.

However, not all gestures have the same impact on learning. Macedonia et al. (2011) contrasted brain activations in response to words encoded with iconic versus meaningless gestures. The authors found premotor activation only for the former, whereas the latter caused an activation in a network associated with the cognitive control. Altogether, these results suggest that the motor component per se is not sufficient to account for the memory advantage; rather, word meaning should be mapped onto the motor program in order to obtain the expected advantage. In addition, a recent experiment confirmed the causal involvement of the motor cortex during recall of enacted words (Mathias et al., 2021): repetitive transcranial magnetic stimulation over the primary motor cortex (M1) affected the translation of L2 words learned with sensorimotor-enriched training (gestures), but not that of L2 words learned with only sensory-enriched training (pictures).

As a further proof of the involvement of the motor system in processing language learned through enactment, Repetto et. al (2021) showed that the motor activation originating in the brain also reaches the muscles when participants process words learned through enactment. If the studies reviewed so far explored the representation of words *after* encoding through gestures, little is known about the brain activations *during* the gesture-based encoding phase. To examine this issue, Macedonia et al. (2019) scanned participants’ brain activity as they encoded words in different conditions: visual, audiovisual, and gesture observation. The network of activated areas reflected the progressive enrichment of the stimuli: a basic network engaged in reading was detected during the visual condition, which was enlarged by auditory cortices for the audiovisual condition, and even more so by motor cortices and parietal lobules in the gesture observation condition.

### What are the neural correlates of L2 learning through motor actions?

Similar to enactment, the term *embodied learning* has been used to describe the act of encoding new information by performing actions (James & Bose, 2011; James & Swain, 2011; Johnson-Glenberg, 2017, 2018; Johnson-Glenberg & Megowan-Romanowicz, 2017). Importantly, the level of embodiment is thought to depend on how physically engaged the learner feels, as well as the congruency between gestures and the content being learned (Johnson-Glenberg & Megowan-Romanowicz, 2017; Skulmowski & Rey 2018). For example, mathematical (Kontra et al., 2015) and scientific principles (Johnson-Glenberg & Megowan-Romanowicz, 2017; Johnson-Glenberg et al., 2016) have been shown to be better integrated when learned with physical activity as opposed to verbalization alone.

Along similar lines as the gesture studies described above, a handful of neurocognitive studies involving non-gesture action have also examined how the motor system might shape the acquisition of lexical items. In a learning paradigm, Fargier et al. (2012) taught participants novel words in association with visual motor actions compared to abstract animated images. In a post-training session, the authors measured motor activation via neural oscillations while participants processed the novel words. After the first day of training, greater suppression in the mu band (8–13 Hz), a frequency band putatively associated with motor activation, emerged for words learned with actions compared to the control condition. Unexpectedly, following the second day of training, mu desynchronization was distributed over fronto-central areas. The authors argued that areas activated by both motor and linguistic processing were hence confined to a *convergence zone* between motor and language structures (i.e., more frontal regions and not the central parietal areas that are thought to subserve sensorimotor activity). This outcome was well in line with mu-rhythm dynamics found for both L2 and L1 action word processing in bilingual subjects (Vukovic et al., 2014). In a similar study performed by Bechtold et al. (2018), participants learned novel names for novel tools through either visual or manual exploration. As expected, results revealed greater mu and beta frequency band suppression post-training for words learned through haptic manipulation. However, non-tool related familiarized pseudowords showed a similar pattern. The authors suggested that differences in mu and beta suppression during the processing of the learned words may have been a result of the suppression of motor activation for processing words that only have visual features. These studies are novel in their attempt to provide neurocognitive evidence of how motor stimulation during novel word encoding affects the representation of these words. However, none of them provide clear evidence that words learned with action directly reactivate sensorimotor information, nor that embodied learning leads to improved word encoding. This latter issue was more recently tackled by involving TMS of motor areas in conjunction with hand action-based word learning in Virtual Reality environment, which showed causal involvement of the motor cortex in action word acquisition and even indicated rapid wide-spread microstructural changes in the language system linked to the motor cortex function (Vukovic & Shtyrov, 2019; Vukovic et al., 2021).

#### Future directions

Within the framework of embodied semantics, the above results point to a need for studies that further investigate how both gestures and actions support L2 learning. Most of the imaging studies have shown the network of areas activated during the retrieval of words learned with different degrees of enrichment; less is known about the brain activity during encoding in progressively enriched conditions. The study by Macedonia et al. (2019) explored brain activations only during gesture observation, while gesture or action execution requires different experimental setups. Future studies could complement the extant knowledge in many ways: for example, researchers could use different methods (e.g., functional Near-Infrared Spectroscopy) to investigate brain activity while words are encoded in dynamic experimental settings where participants are not required to stay still, as is the case for fMRI settings, in order to pinpoint the neural correlates of encoding through enactment. In addition, EEG studies could complement the extant knowledge with specific information on the time-course of brain activation changes associated to different encoding conditions. Finally, greater ecological validity in experiments examining the effect of body engagement, whether in the form of gestures or actions, could lead to a deeper understanding of how motor processes affect language learning. Specifically, the combination of neural measures and Virtual Reality, as will be further explored in the final section of this consensus paper, is a promising methodology that allows for more immersive, closer-to-life learning set-ups in which to explore motor-semantic interactions during language learning.

## How Does Object Modality Affect Associative Word Learning and Modulate the N400?

Humans experience the world through sensory input, and language allows us to communicate these experiences to one another, including naming and describing visual, auditory, olfactory, haptic, and gustatory objects and events. Recent research has provided insights into the distribution of nouns pertaining to the allocation of perceptual modalities in vocabularies across several languages (e.g., Chedid et al., 2019; Chen et al., 2019; Lynott et al., 2020; Miklashevsky, 2018; Morucci et al., 2019; Speed & Majid, 2017; Vergallito et al., 2020; Winter et al., 2018). However, research on word learning typically focuses on the acquisition of and mapping of words onto visual objects (e.g., Friedrich & Friederici, 2008, 2011; Horst & Samuelson, 2008; Junge et al., 2012; Smith & Yu, 2008; Taxitari et al., 2019; Werker et al., 1998). It is known that sensory modality not only impacts the outcome of learning, but also the parameters of learning, for example directionality of preference (novelty effects vs. familiarity effects) and age (Emberson et al., 2019; Thiessen, 2010); yet, this has not often been considered in the context of language acquisition. Thus, differences between learning words for visual objects and other object modalities, such as auditory objects, are not well known. An understanding of how word learning takes place for other modalities is important, as perception is the key factor of embodied and embedded cognition within a situated cognition framework (cf. Venter, 2021). Recent research in infants has suggested that 10-month-olds are able to map labels onto auditory objects (i.e., environmental sounds) in a similar way to mapping labels onto visual objects (Cosper et al., 2020). Moreover, recent research has also provided evidence that not only the modality of the object (visual vs. auditory) but also the temporal synchrony of stimulus presentation can also have an effect on learning in both ERP and behavioral measures (Cosper et al., 2022).

The sensory modality of processing has been known to have an effect on memory. This can be seen specifically in recognition memory, where, for example, the recognition for visual objects is found to be superior to that of auditory objects (Cohen et al., 2009, 2011; Gloede & Gregg, 2019). Furthermore, short-term memory and delayed recognition in the visual and tactile modalities has also been shown to be superior to that in the auditory modality (Bigelow & Poremba, 2014). However, short-term memory differences seem to diminish and memory recall becomes more similar when the complexity of stimuli in the auditory and visual modalities are closely matched (Visscher et al., 2007). Thiessen (2010) identified two constraints to modality effects on word learning in adults and infants: the *developmental constraint*, which refers to differences in auditory stimulus processing between infants and adults process the auditory stimuli differently, and the *stimulus constraint*, implying that characteristics of word-object stimuli are processed differently according to modality.

In order to acquire the meaning of a word, the object and its label must first be mapped onto one another. This relationship can be formed in two basic, yet different, ways, in which the outcomes are not necessarily mutually exclusive: by means of referential relationships, such as hypothesis testing (cf. Xu & Tenenbaum, 2007) or by means of associative relationships (e.g., Bergelson & Aslin, 2017; Bergelson & Swingley, 2012; Friedrich & Friederici, 2011; Sloutsky et al., 2017). In the case of word learning for auditory objects, the cognitive mechanisms of maintaining auditory short-term memory are important considerations for the outcome of learning. Baddeley (2012) gives a review of the *phonological loop*, an auditory working memory maintenance system reliant on vocal or subvocal rehearsal. The phonological loop is said to not only be utilized for auditory-verbal stimuli, but also for auditory-nonverbal stimuli (Baddeley, 2012). However, more recent research has provided an alternative system of working memory maintenance for auditory-nonverbal stimuli, namely a cognitive mechanism similar to visual imagery – *auditory imagery* – which details that auditory-nonverbal information may be maintained in working memory by imaging a sound (Soemer & Saito, 2015). Albeit there are differences in the two mechanisms underlying working memory in the auditory modality, it could be that both are necessary in auditory word learning, as both auditory-verbal and auditory-nonverbal information is present in such learning situations in the form of spoken words and auditory objects, for example, environmental sounds. This notion has been seen in adult learners, but needs to be further explored and excplicitly manipulated in an experimental setting in order to fully understand how the pholological loop and auditory imagery are affected by object modality in word learning (cf. Cosper et al., 2022).

The ERP method can be applied in order to provide insights into the neural underpinning of learning, along with additional behavioral measures such as accuracy and reaction times obtained through an active learning paradigm (McLaughlin et al., 2004). We already mentioned N400, as one of the most important components in linguistic ERP research; it is not surprising that it has also been used to address the learning processes. In addition to the classical sentential contexts, the N400 has also been used to measure word learning in isolation (for reviews, see Junge et al., 2021; Kutas & Federmeier, 2000, 2011). In addition to spoken or written words, the N400 component can also be used to measure processing of non-linguistic material, such as tones, music, and environmental sounds (Cummings et al., 2006; Koelsch et al., 2004; van Petten & Rheinfelder, 1995), indicating that the N400 can be used as a measure of violated expectation with both auditory-verbal and auditory-nonverbal stimuli. As such, the N400 component can be used to measure learning in associative word learning paradigms during and after learning with both auditory and visual objects paired with spoken pseudowords.

Recent empirical evidence has been provided that infants are able to map words onto auditory objects (i.e., environmental sounds) in a similar way as they do for visual objects (Cosper et al., 2020); however, first evidence provides insight in that auditory associative word learning is less effective than visual associative word learning (Cosper et al., 2022). Despite these findings, there is still a gap in the literature on how the full spectrum of perceptual modality features affects word learning and emodied and situated cognition of language processing. Nonetheless, one can speculate about the possibility of object modality affecting associative word learning and, in turn, modulating the N400 in adults. The functional interpretation of the N400 effect is generally described as either a process of spreading activation (e.g., Holcomb, 1988; Kiefer, 2002; Lau et al., 2008) or semantic integration (e.g., Bentin et al., 1993; Kutas & Federmeier, 2011). However, as mentioned above, there are other functional interpretations of the N400 effect, which include factors such as predictability (semantic-level), plausibility (sentence-level), and similarity (low-level semantic relationship, based on co-occurrence; Nieuwland et al., 2020). More recent research also shows that the latency of the N400 (or N500, cf. Schöne et al., 2018) is sensitive to tension and stress in processing personally relevant stimuli (Schöne et al., 2018; Carretie et al., 2005). As such, factors beyond semantic integration or spreading activation can affect the latency of the N400 effect; thus, it is possible that object modality also can affect the latency of the N400 effect (cf. Cosper, 2020; Cosper et al., 2022). It is also important to note that the topological distribution of the N400 can also differ across studies, which can be influenced by factors such as stimuli-specific processing demands, vocabulary, and age (for a review in infants and young children, see Junge et al. 2021); however, this section focuses on and highlights the effects on latency. Based on these alternative functional interpretations of the N400 component, we can consider how object modality may also reflect functional differences in the processing, learning, and mapping of labels onto objects of various modalities.

In order to consider object modality in terms of functional differences pertaining to associative word learning, one must first consider how modality affects processing, memory, and word acquisition both in isolation and taken together. Importantly, the N400 component itself is an effective measurement of processing and learning with non-linguistic auditory stimuli (Cummings et al., 2006; Koelsch et al., 2004; van Petten & Rheinfelder, 1995). It is further well known that the N400 component can also measure violations of (semantic) expectation in line drawings (cf. Kutas & Federmeier, 2000). Thus, the N400 component itself is highly flexible and can be used to measure learning by means of violated (lexico-)semantic expectation in at least the visual and the auditory-verbal as well as auditory-nonverbal modalities.

As briefly presented above, it is known that object modality affects recognition memory, short-term memory, delayed recognition, and memory recall (cf. Bigelow & Poremba, 2014; Cohen et al., 2009, 2011; Gloede & Gregg, 2019; Visscher et al., 2007) as well as statistical learning, extracting regularities from repeated exposure to information input over time, in both infancy and adulthood (e.g., Emberson et al., 2019; Saffran, 2002; Saffran et al., 1996, 1999). Another recent study (Miller et al., 2018) provided evidence that, following a series of training sessions with implicit verbal-tactile association learning, participants exhibited enhanced tactile perception to consistently labeled verbal-tactile pairs compared to those who were trained on inconsistently labeled pairs, thus suggesting a causal relationship between language and perception (see also Schmidt et al., 2019 and the L2 section above). In sum, associative word learning is affected by object modality (visual vs. auditory) and, given the latency costs in processing, recognizing, and recalling visual versus auditory stimuli, the latency of the N400 component in auditory associative word learning may be functionally affected by the modality of the object being labeled.

### Future directions

Although infant associative word learning with visual and auditory objects may be similar, there is much left to be explored and discovered in both infant and adult word learning. Despite modality being known to affect many areas of cognition and the reflection of sensory modality in language and its vocabularies, much of what is known about word learning has been conducted with mapping labels onto visual objects. This can be seen in how mechanisms behind maintaining short-term memory in the auditory modality can be disrupted when both auditory-verbal and auditory-nonverbal information is being maintained simultaneously. In order to better understand how perception and language are linked throughout the span of life, word learning must be expanded into the auditory, haptic, olfactory, and gustatory modalities in a greater capacity, as there are various differences in learning across modalities that differ during development than in adulthood. With an understanding of how perceptual features of object affect word learning, a greater understanding of how perceptual features affect embodied and situated cognition can also unfold.

Furthermore, it is vital that word learning, and associative word learning in particular, be expanded outside of western, educated, industrialized, rich, and democratic societies (WEIRD) societies. Majid et al. (2018) have shown that non-WEIRD languages and populations deviate in their linguistic expression of perceptual modality as compared to WEIRD societies, in which other modality norms in the non-WEIRD languages (e.g., indigenous languages) prevail than the visual modality being the most represented (cf. Chedid et al., 2019; Chen et al., 2019; Lynott et al., 2020; Miklashevsky, 2018; Morucci et al., 2019; Speed & Majid, 2017; Vergallito et al., 2020; Winter et al., 2018). Moreover, with reference to individual experience, a greater understanding of the effect of modality on word learning could be explored in blind, deaf or even individuals with synesthesia. In further studying these populations and languages, a broader picture of situated and embodied cognition, as it pertains to language acquisition and processing, and the relation between perception, language, and situated cognition will begin to form.

## What Can Virtual Reality Bring to Embodied Language Processing and Learning Studies?

When it comes to how language is processed and learned, theories of embodied and situated cognition give a great deal of importance to physical contexts. This poses a major challenge for neurocognitive studies in these fields, as it calls for more multimodal and close-to-life experimental protocols that are more ecologically valid while still allowing for strict experimental control (Tromp et al., 2018; Peeters, 2019). In this context, VR approaches may provide an important tool to achieve this balance. VR has been said to eliminate the spatial divide between stimulus and participant (Peeters, 2019). Clearly relevant for embodied language processing and learning research, VR provides participants with more ecologically valid, interactive and immersive environments, thought to better engage the sensorimotor system and elicit real life responses (Bohil et al., 2011). Furthermore, while language studies are generally constrained to focus on a single modality (e.g., speech), VR allows for the observation of how different modalities (e.g., speech, body movements, facial expression) interact with each other – as is the case in real-life communication – in rich, closer-to-life environments (Peeters, 2019). Finally, immersive VR protocols allow participants to engage in semi-natural actions, which should lead to results that can generalize to real life (Peeters, 2019).

So far, very few studies have combined VR and cortical measures to investigate embodied language processing. Tromp et al. (2018) validated the use of VR to investigate language processing by measuring EEG while participants wore a head-mounted VR display, immersed in a virtual restaurant. As they listened to a sentence (“*I just ordered this salmon*”), participants saw a virtual object that either matched (*salmon*) or mismatched (*pasta*) the object in the sentence. As expected, a match-mismatch N400 effect emerged when items were incorrectly labeled orally, compared to correctly labeled items. This was considered a proof of concept for combining EEG and immersive VR to observe neurocognitive language processing. Within an embodied semantics perspective, Zappa et al. (2019) measured cortical motor activation during action verb processing in a Cave automatic virtual environment (an immersive VR environment that surrounds users while allowing them to see their own body). The study focused on changes in the mu (8–13 Hz) and beta (20–30 Hz) frequency bands, associated with motor activity, as participants performed a Go-Nogo task. Participants heard action verbs and, for Go trials, performed a corresponding action on a virtual object. Mu and beta band suppression was found for both Go and No-Go trials. Importantly, mu suppression emerged 400–500 ms after action word onset, associated with lexical-semantic processing, and more so for Go trials, indicating an interaction between motor and linguistic processes. Whereas L1 studies have used VR to examine language processing, a few experiments have taken advantage of this technology to explore L2 learning. In a longitudinal fMRI study, Legault et al. (2019) taught L2 words to novice learners using the Second Life gaming platform virtual environment, compared to a control group that learned via picture-word association. For both groups, results revealed increased cortical thickness and gray matter volume in regions implicated in a language control network after training. Furthermore, within this network, L2 training in the picture-word association group led to greater cortical thickness in the right inferior frontal gyrus, whereas training performance in the VR group was positively correlated with cortical thickness in the right inferior parietal lobule (associated with superior L2 proficiency, Mechelli et al., 2004). Also, for the VR group, accuracy in the delayed retention post-training was positively correlated with cortical thickness in the right supramarginal gyrus. These results indicate that training in virtual environments leads to rapid cortical changes that differ from those found after picture-word association learning. Also, VR learning is thought to have led to a stronger engagement of the inferior parietal lobule in immersive and interactive L2 learning, as it stimulated embodied processes (Li et al., 2020).

Using a different approach, in a TMS study Vukovic and Shtyrov (2019) examined embodied semantics in a VR setting by interfering with participants’ M1 during novel word learning in order to test whether this would affect verb encoding. An interactive VR computer game was used to teach participants novel labels for object nouns and action verbs as they manipulated virtual objects. Theta-burst TMS was applied to participants’ M1 prior to learning and, as predicted, they were less successful at encoding novel verbs compared to nouns when the hand area of the left M1 was stimulated. Applying theta-burst TMS to the M1 was thought to prevent a motor trace from forming for verb labels, suggesting that motor cortex activity was involved in the early stages of word encoding. Furthermore, the same VR-TMS design combined with diffusion kurtosis imaging (DKI) showed a range of microstructural changes taking place in the brain over the short learning session (Vukovic et al., 2021). Generally speaking, the above-described language processing and learning studies illustrate that VR allows for the manipulation of naturalistic movement and environments and can be combined with cortical measures to successfully examine embodied semantics.

A completely new field of research is concerned with the investigation of the role of the virtual action (VA) on language learning and comprehension. A VA is an action performed within a virtual environment, exploiting VR technology. Experiencing a virtual world immersively (i.e., by means of a head-mounted display or a Cave automatic virtual environment) gives users a subjective feeling of agency. Specifically, immersive VR allows us to represent our movements from the first-person perspective and to feel like agents of the actions we perform. Therefore, VR users can have the subjective feeling of performing a VA either by seeing their virtual body parts performing an action (e.g., the virtual hands grasping an object) or by perceiving changes in the optical flow consistent with that action (e.g., users can feel as if they are virtually walking when presented with a coherent change in the optical flow of the virtual environment). It should be noted that a VA can have a real counterpart, i.e a real action performed by the individual, which either matches the virtual one or not (the user can point in a given direction with her real hand and, at the same time, see her virtual hand perform the same action – match; or the user can press a button with her real hand and see the virtual hand pointing to something – mismatch). In some cases, the VA can also have no real counterparts (e.g., the user is still but sees herself walking into the virtual environment). A few studies have begun to shed light on the cortical underpinnings of VA (Adamovich et al., 2009; Holper et al., 2010). According to their findings, the observation, imagery, and execution of actions with real-time virtual feedback share common neural substrates located within the observation-execution network. Carrieri et al. (2016) investigated the cortical activity during a demanding hand controlled task in VR, and observed a ventrolateral prefrontal cortex involvement, compatible with the recruitment of attention resources needed to accomplish the task. A VA performed with the legs (virtual walk) was investigated by Wagner et al. (2014), who manipulated the visual feedback provided within the virtual environment. They found that during the virtual walk (with coherent visual feedback in the 1^st^ and 3^rd^ person point of view) premotor and parietal areas showed increased activity compared to the conditions in which the visual feedback was unrelated to the motor task, or in which it consisted of a mirror-image of a VR avatar. Taken together, these results clearly indicate that VA can modulate the cortical motor system.

The impact of VA on language processing has been investigated by a few studies using basic virtual environments (Repetto, Cipresso, et al., 2015; Repetto, Colombo, et al., 2015), which demonstrated that a VA performed with a specific body part (e.g., legs) can facilitate semantic comprehension of verbs describing actions performed with the same effector (Repetto, Cipresso, et al., 2015). Similarly, learning L2 words could be improved by performing a VA with the effector matching that described by the action verb to be learned (Repetto et al., 2015).

### Future directions

Considering, on one hand, the behavioral evidence and, on the other hand, neurophysiological studies run in virtual environments, future studies could exploit the capabilities of VR/VA to improve language learning and rehabilitation. Indeed, as pointed out in a seminal paper by Pulvermuller and Berthier (2008), individuals could try to stimulate language through the action system, and in particular VA could be used to provide language training in safe yet controlled settings. VA-based protocols could be useful for both L2 learning and aphasia rehabilitation. In both cases, the acquisition or re-acquisition of words could be supported by concomitant VA execution. Indeed, in the field of L2 learning, previous studies have already shown the benefit of self-performed action while encoding novel words (see previous section). Thus, the switch to a VA from a real one could offer the opportunity to increase the range of possible actions (including also those that are, for instance, impossible or even dangerous in a rehabilitative setting). In addition, the use of VA could extend the training portability in all those contexts wherein it is not easy to move due to environmental or social constraints. Similarly, the rehabilitation training for naming disabilities could benefit from the association of a VA along with verbal stimuli: previous research, inspired by the embodied approach, has implemented training based on action observation (Bonifazi et al., 2013). However, it has been demonstrated that cortical excitability is greater for actions experienced from the first-person perspective (Maeda et al., 2002), therefore the visualization of VA could be even more beneficial than traditional action-observation protocols. Furthermore, aphasic patients with concomitant motor deficits, who are unable to move efficiently, could benefit from VA-based trainings that would give them the impression of moving the affected limb(s).

## Conclusion

According to situated and embodied theories (including embodied, embedded, extended, enacted, and grounded cognition approaches), language representation is intrinsically linked to our interactions with the physical world. The initial rivalry between embodied and amodal theories has given way to a more flexible approach that aims to determine *when* and *how* perceptuomotor mechanisms are involved in language processes, alongside modality-independent processes. This consensus paper is not an exhaustive review of embodiment studies but rather addressed carefully selected questions by presenting seminal research as well as recent methodological developments that can point to future directions in the research field of embodied and situated language processing.

In the area of language processing, we presented evidence based predominantly on ERPs/ERFs as well as oscillatory markers that, at least in some contexts, suggests that motor and perceptual information comprised in language representation rely on brain resources partially overlapping with motor and perceptual experience. We are currently facing the challenge of the functional interpretation of these markers. Future research investigating, e.g., the laminar profile of the underlying neural resources seems promising to examine how the same resources can subserve both the physical experience and semantic processing. Further, the comparison between abstract and concrete word processing was shown to unveil fundamental differences in their grounding. A crucial next step is to systematically explore task-dependent, situated grounding along the concreteness continuum. We showed that semantic and cross-domain relations lead to differential priming effects, which can reveal organizational principles interconnecting the multiple dimensions of embodied word representations. Future research should focus on individual and situational differences in these effects as well as on disentangling whether cross-domain priming relies on an overlap in general cognitive mechanisms vs. specific representational content. In the area of language learning, we showed that word acquisition based on gestures and other motor actions reveals a supporting and at least partially causal role of embodiment in acquiring language representations. Here, future research might focus on greater body engagement and manipulation of the environment via VR to enhance ecological validity as well as target the role of perspective-taking. We provided evidence that object modality affects the N400 in adult word learning in a situated manner. The generalizability across perceptual modalities and culturally diverse societies is an important future step. Finally, we argued for the value of VR and VA as tools to support and modulate language learning and rehabilitation, providing highly controlled, close-to-life set-ups supporting language (re-)acquisition in previously inaccessible situations.

The puzzle pieces presented above point to common future directions in investigating the brain signatures of embodied and situated language processes. Our research field has greatly benefitted from fundamental research testing well-defined hypotheses by following a reductionist approach to multimodality (e.g., motor vs. visual information in learning/processing language entities from distinct categories) or semantic multidimensionality (e.g., the concrete vs. abstract distinction). We value this seminal research, as it formed and informed a dense theoretical network, which we can now use as a launch pad to aim higher and achieve external validity based on more complex research designs with more sophisticated as well as more ecologically valid methods. Importantly, new acquisition and analysis methods based on ever-growing computing capacities now call for acknowledging the multimodality, multidimensionality, flexibility and idiosyncrasy in embodied and situated language processes. These dimensions are determinant in probing embodied and especially situated language processes in the future, as they can potentially explain many of the discrepant findings of studies following a reductionist approach.

The red thread through the sections presented above is a consensus on a basic understanding of embodied, situated language processes: these do not require that (primary) sensorimotor areas be automatically and causally involved independently from stages of language learning, representation, and processing or individuals or tasks and contexts (see also Barsalou, 2020). In contrast, brain signatures of embodied and situated language processes are flexible, as their evolutionary rationale with the primary goal to allow an efficient online processing of language calls for this flexibility in the face of our ever-changing environment. The complexity and versatility of situated embodied language processes thus cannot be reduced to a dyadic (on/off) character as suggested by the concept of all-or-nothing causality and automaticity. The *replication crisis* (Kellmeyer, 2017; Laws, 2016; Zwaan et al., 2017) luckily forced research in this field to tackle this problem by running large-scale studies investigating embodied phenomena in multiple different tasks (e.g., ManyBabies Consortium, 2020; Morey et al., 2021; Pavlov et al., 2021), by taking into account cultural diversity (Bettinsoli et al., 2021), and providing research guidelines to make ERP research more consistent in design and reporting (e.g., Paul et al., 2021; Šoškić et al., 2021; Styles et al., 2021).

With this development, research in our field can now move from a focus on internal validity, which favored reducing the dimensions of our investigations, to a focus on external validity, which now begins to favor more true-to-life, complex research designs. In this sense, future research can examine multidimensionality and multimodality in language processes rather than breaking them down to, for example, dichotomous formats. We can move from investigating isolated semantics to the broad variety of mechanisms and interactions involved in contextual language processes. Furthermore, we can use VR to enhance external validity by incorporating more true-to-life movements and perceptual experience in a still controlled laboratory setting. Instead of asking *whether* language processing is embodied, we can ask *how* and *under what circumstances* perceptuomotor brain areas support conceptual processing. In this regard, a special focus on the functional interpretation of long-standing markers of embodied language processes like different ERP/ERF components, neural oscillations and their time course as well as neuromodulation methods that can show causal brain-behavior relationships will accompany us throughout the next decades of research on the brain signatures of embodied and situated language processing. Overall, this consensus paper delivers a glance at the state of the art regarding different factettes of the research around embodied and situated language processes. Our hope is that it will help other researchers to identify where their research stands in relation to what has been examined and *how* it has been examined. Specifically, we hope that theoretical and practical implications from some of the fields we have discussed (e.g., priming studies, VR studies, etc.) could inspire other fields within embodied & situated language processes research.

## Author Notes

Correspondence regarding the content of specific sections can be addressed to the following authors (in the case of multiple authors in a section, authors listed in alphabetical order):

What kind of neural evidence is there for embodied semantics? Piermatteo Morucci (piermatteo.morucci@gmail.com) and Valentina Niccolai (valentina.niccolai@hhu.de). How are abstract and concrete words grounded? Maria Montefinese (maria.montefinese@gmail.com). What can semantic priming tell us about embodied word representations? Laura Bechtold (laura.bechtold@hhu.de) and Anastasia Malyshevskaya (malyshevskaya.com@gmail.com). What can embodied L2 learning teach us about the role of embodiment in language representation? Claudia Repetto (Claudia.Repetto@unicatt.it) and Ana Zappa (ana.zappa@univ-amu.fr). How does object modality affect associative word learning and modulate the N400? Samuel H. Cosper (samuel.cosper@uni-osnabrueck.de). What can virtual reality bring to embodied language processing and learning studies? Claudia Repetto (claudia.repetto@unicatt.it) and Ana Zappa (ana.zappa@univ-amu.fr).
